# Supplementary material for: Integrating long noncoding RNAs and mRNAs expression profiles of response to Plasmodiophora brassicae infection in Pakchoi (Brassica campestris ssp. chinensis Makino)
Source: PLoS One. 2019 Dec 5;14(12):e0224927. doi: 10.1371/journal.pone.0224927 (PMC6894877; doi:10.1371/journal.pone.0224927)
Supplement: S1 Table — (DOC) [file pone.0224927.s001.doc]

**S1 table. Primers used in this study**

| **Gene Symbol** | **Primer** | **Sequence** | **Product length(bp)** | **Ta(℃)** |
| --- | --- | --- | --- | --- |
| TUB6 | F | AGTGGACGAGCAGATGATAA | 94 | 60 |
|  | R | TATGTCACAGACGCTTGACT |  |  |
| evm.model.BraA03002406 | F | TCTCAACCTTAACCTCTTAGGC | 100 | 60 |
|  | R | CGGTCTTACGCTATTCATCCA |  |  |
| evm.model.BraA01004402 | F | TACCGGATACTTTGTATGGTCT | 116 | 60 |
|  | R | CTTTCTCATATCGTGTGAGGTT |  |  |
| evm.model.BraA07003532 | F | CAGCATTTCGATCTCTCTCAG | 98 | 60 |
|  | R | CTTCTCACGCAAGACACTC |  |  |
| evm.model.BraA03003380 | F | TATGTGCCTTTGCTGCCA | 114 | 60 |
|  | R | AGCAATACGCACTTACTCG |  |  |
| evm.model.BraA01000469 | F | GGCCCTATGGAGAGAACATC | 109 | 60 |
|  | R | CATGTGTTGGAACCATAATCGT |  |  |
| evm.model.BraA02003020 | F | ACTTACAACAACGGCGAC | 92 | 60 |
|  | R | CCACCGGGTTTCATTGATT |  |  |
| evm.model.BraA02003539 | F | ATCCTCTGTTCATGGATACGA | 86 | 60 |
|  | R | CACCCATTGTTCCTGAAGC |  |  |
| evm.model.BraA03001724 | F | GTACGAAGCACAGCTTTCAA | 86 | 60 |
|  | R | ACTCAGTGAAGGGAAGGT |  |  |
| evm.model.BraA02000287 | F | TCCATTTCAGCATATGGGCTA | 102 | 60 |
|  | R | CCTCCTTGTTTGCCACAATC |  |  |
| evm.model.BraA10000326 | F | CAGAGCAGCGAACCCATTA | 105 | 60 |
|  | R | AAGGCGGTGGATGAATTG |  |  |
| evm.model.BraA03002405 | F | TGGTAACATTGTGACACAAGG | 96 | 60 |
|  | R | ATGAAAGAGTCACGGGTATAGA |  |  |
| evm.model.BraA06004963 | F | TTGAGACTACAGCCTTCGAT | 102 | 60 |
|  | R | AGGGAAGAGTTTAGCTCCA |  |  |
| evm.model.BraA02001770 | F | GGATGCGACGAGCCTTTA | 107 | 60 |
|  | R | ACGTCGATCCTCCGAAGTA |  |  |
| evm.model.BraA09002494 | F | ATATCTGGACAAGACAAAGCAC | 100 | 60 |
|  | R | GTGATGAGAATCTCTCGGAATG |  |  |
| evm.model.BraA04001958 | F | GAAGCAGAGCTTCAAGACAT | 110 | 60 |
|  | R | GAGTCCGTGTCCTTCATCTTAC |  |  |
| evm.model.BraA03003637 | F | TACCGGATACTTTGTATGGTCT | 118 | 60 |
|  | R | GAGTCCGTGTCCTTCATCTTAC |  |  |
| TCONS_00050767 | F | AGCTCCTCTTCTTCTTCTATCT | 87 | 60 |
|  | R | CCGGTATTAATTGTATGGCGT |  |  |
| TCONS_00007793 | F | GGATAGGAATCAATCTTCGCC | 102 | 60 |
|  | R | CCTCATCTGGAACTGCTTCA |  |  |
| TCONS_00036594 | F | TACTTTCTGAGTGGTGCCC | 147 | 60 |
|  | R | CAGCAGCAGTAAGAGAAGC |  |  |
| TCONS_00051667 | F | AGTAAGAGAAGAAATGGCAGG | 100 | 60 |
|  | R | GTCACACTCCGTGTTGTAG |  |  |
| TCONS_00014032 | F | GGCAAGTCATCAGAGGCAAA | 101 | 60 |
|  | R | AGAGAATCAACACATCACCG |  |  |
