# Supplementary material for: Integrating long noncoding RNAs and mRNAs expression profiles of response to Plasmodiophora brassicae infection in Pakchoi (Brassica campestris ssp. chinensis Makino)
Source: PLoS One. 2019 Dec 5;14(12):e0224927. doi: 10.1371/journal.pone.0224927 (PMC6894877; doi:10.1371/journal.pone.0224927)

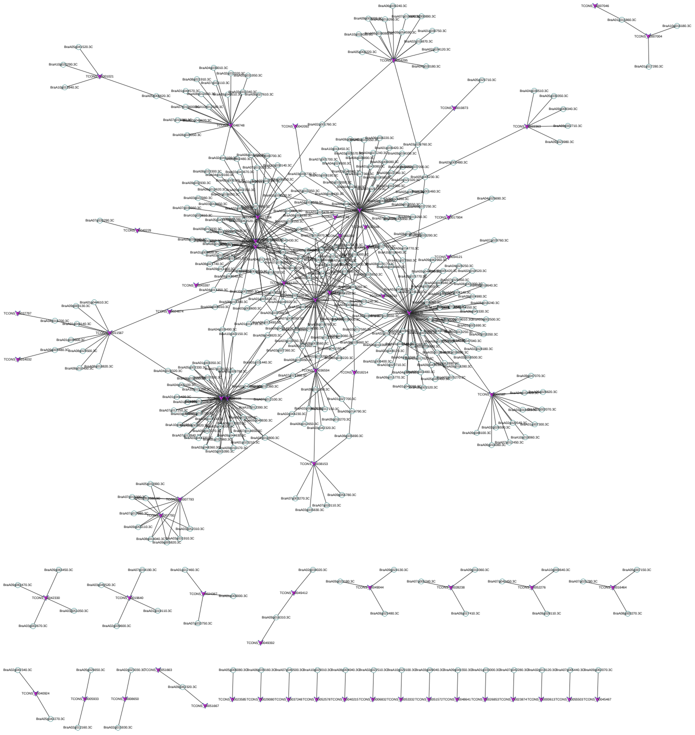

Supplement: S1 Fig — The arrow and circle nodes denote lncRNA and mRNA, respectively. Each gray edge denotes a potential target relationship between a gene or lncRNA. (PDF) [file pone.0224927.s006.pdf]
